# The frequency of Raynaud’s phenomenon in patients with methylenetetrahydrofolate reductase gene mutation and hyperhomocysteinemia

**DOI:** 10.3906/sag-1903-206

**Published:** 2019-10-24

**Authors:** Kadir Serkan YALÇIN, Ali KOŞAR

**Affiliations:** 1 Department of Internal Diseases, Lokman Hekim Ankara Hospital, Ankara, Turkey; 2 Department of Hematology, Lokman Hekim Ankara Hospital, Ankara Turkey

**Keywords:** Raynaud’s phenomenon, hyperhomocysteinemia, methylenetetrahydrofolate reductase, mutation

## Abstract

**Background/aim:**

Raynaud’s phenomenon (RP) is not a rare health problem; global prevalence is about 3%–20%. Etiology and pathophysiology of this pathology has not been clarified. There are many precipitating factors resulting in RP. Hyperhomocysteinemia resulting from methylenetetrahydrofolate reductase (MTHFR) gene mutation may have a role in its etiology. The aim of this study was to observe the frequency of RP in patients with MTFHR gene mutation and hyperhomocysteinemia. Possible relationships among vitamin B12, folic acid, complete blood count (leukocytes and platelets), and c-reactive protein levels and RP were also analyzed.

**Materials and methods:**

A total of 388 patients admitted to the internal medicine, hematology, and obstetric clinics of a university hospital between January 2012 and April 2013 ranging in age from 21 to 83 (mean age 38.16 ± 13.1) were enrolled in the study. Eighty-five (21.9%) of the patients were male and 303 (78.1%) were female. MTHFR gene mutation was analyzed in 388 patients; 52 (13.4%) were homozygous, 275 (70.9%) were heterozygous, and 61 (15.7%) were found to be negative for the MTHFR gene mutation and accepted as a control group. Vitamin B12, folic acid, complete blood count (leukocytes and platelets), and c-reactive protein levels were also analyzed.

**Results:**

Homocysteine levels were higher in both heterozygous and homozygous groups (P < 0.05). RP was more frequently observed in patients with elevated homocysteine levels (P < 0.05; X2 = 14.51). There was no significant relationship in other parameters studied.

**Conclusion:**

RP was more frequently observed in the groups with the MTHFR mutation and hyperhomocysteinemia. Serum homocysteine levels in patients with RP may be helpful for diagnosis.

## 1. Introduction

Raynaud’s phenomenon (RP) is paroxysmal reversible ischemia resulting from abnormal arterial vasospastic response due to recurrent cold or emotional stress in the extremities or peripheral parts of body [1]. Vasospasm causes diminished arterial blood supply and paleness will be observed, deoxygenation of hemoglobin will result in a dark-blue color change reflecting cyanosis; finally, acidosis will trigger vasodilatation and hyperemia and the color will change to red [2]. When there’s no underlying condition, RP is called primary Raynaud’s phenomenon (PRP). RP may also be seen in some systemic diseases and conditions (e.g., scleroderma, systemic lupus erythematosus, mixed connective tissue disease, Sjögren’s syndrome, end-stage renal disease, hand–arm vibration syndrome, antimigraine drugs, beta blockers, cyclosporin, hypothyroidism, pheochromocytoma, paraneoplastic syndrome, Buerger’s disease), which is called secondary Raynaud’s phenomenon (SRP) [3]. Various systemic factors are involved in the pathogenesis of RP; increased alfa 2 adrenergic receptor activity, especially under cold exposure, or endothelin-1 activity may result in vasoconstriction [4]; decreased nitric oxide (NO) [5] or calcitonin gene related peptide (CGRP) [6] may cause ineffective vasodilation; injury to endothelium because of increased oxidative stress may result in improper endothelial responses; platelet activation, decreased fibrinolytic activity [7], decreased red blood cell flexibility through arterioles [8], and increased intravascular viscosity [9] may also have unfavorable effects on blood flow. The prevalence of RP is reported to be 3%–20% worldwide, and it is more frequently seen in colder climates and in females [10,11]. Prevalence in Turkey was reported to be 5.9% in one study [12] and 3.6% in another [13].

The 5,10-methylene tetrahydrofolate is the major protein acting as a carbon donor in the remethylation of homocysteine to methionine reaction. It is converted to 5-methyl tetrahydrofolate by the methylene tetrahydrofolate reductase (MTHFR) enzyme. Metabolism of homocysteine requires vitamin B6, B12, and folate as cofactors [14,15]. Several mutations and polymorphisms in MTHFR enzyme genes will result in defective or diminished enzyme function [16,17]. MTHFR enzyme deficiency will cause higher homocysteine levels. A slight decrease in MTHFR enzyme activity may result in serious outcomes, particularly in arterial diseases such as peripheral neuropathy, due to microangiopathy, stroke, thrombosis, and coronary artery disease [18,19].

Hyperhomocysteinemia is found to be associated with vascular pathologies by causing endothelial dysfunction [20]. Hyperhomocysteinemia may be related to vitamin B6, B12, and folate deficiencies; supplementation of folic acid may improve homocysteine levels [21].

There is only one study reported in the literature that examined homocysteine concentrations in patients with MTHFR gene mutations and primary or systemic sclerosis-associated RP [22].

The aim of this study was to compare patients experiencing RP with normal MTHFR enzyme activity to patients with mutations, and to evaluate the relationship between MTHFR enzyme mutation, homocysteine levels, and frequency of RP. Other purposes were to inspect any possible relationships among vitamin B12, folic acid, leukocytes and platelets (by complete blood count), c-reactive protein levels, and RP.

## 2. Materials and methods

This study was approved by the local ethics committee (38918275/0052). Informed written consent was obtained from all of the patients participated in the study. This is a prospective observational cohort study conducted between January 2012 and April 2013 on patients who were admitted to internal medicine, hematology, and the gynecology and obstetrics clinics of a university hospital for spontaneous abortus, recurrent abortus, varicose veins, or deep venous thrombosis, and whose MTHFR gene mutations were analyzed simultaneously with other biochemical parameters [antinuclear antibody, vitamin B12, folic acid, leukocytes and platelets (by complete blood count), c-reactive protein levels after 12 h of fasting]. Homocysteine levels above 12 mmol/L were considered high. The 677CT and 1298AC mutations were inspected by real-time polymerase chain reaction (PCR) testing. DNA isolation from peripheral blood was performed with a QIAGEN QIAamp DNA blood kit (QIAGEN N.V., Venlo, the Netherlands).

All of the patients included in the study were questioned for the presence of RP. Existing RP was diagnosed after an icy water stress test. Before testing, patients’ finger temperatures were recorded and confirmed to be above 30 °C. Patients were requested to hold both hands in a container filled with water and ice cubes for 30 s; finger temperatures were recorded at 5 min intervals. Patients whose finger temperatures were still below 30 °C after 10 min were accepted as having RP. Exclusion criteria are shown in Table 1.

**Table 1:** Exclusion criteria of the patients.

1. Alcohol consumption, smoking, any kind of drug abuse
2. Anticoagulant medication usage for any reason
3. Patients with systemic or hematological disorders that may lead to thrombophilia (diabetes, chronic kidney disease, systemic lupus erythematosus, rheumatoid arthritis)
4. The diagnosis of malignancy or being treated because of a malignancy
5. Hypothyroidism
6. Age; people younger than 20 years or older than 85 years
7. Did not want to participate in the study
8. Vitamin supplementation usage
9. Antinuclear antibody positivity
10. Elevated erythrocyte sedimentation rate and c-reactive protein levels

Patients with normal MTHFR enzyme activity were accepted as the control group and heterozygous– homozygous mutation-positive individuals constituted the study groups.

Three hundred and eighty-eight patients were included in the study. The ages of patients ranged from 21 to 83 years, with a mean age of 38.16 ± 13.1 years. Eighty-five (21.9%) of the patients were male and 303 (78.1%) were female.

MTHFR enzyme gene mutation was analyzed in all patients; 61 (15.7%) were found to be negative for the MTHFR mutation and were accepted as the control group. Two hundred and seventy-five patients (70.9%) had the heterozygous mutation and 52 patients (13.4%) were found to have the homozygous mutation.

### 2.1. Statistical analysis

SPSS for Windows 20.0 statistical software package (IBM Corp., Armonk, NY, USA) was used for statistical analysis of the data. The results of all the parameters of the cases was given as the mean +/- standard deviation. In the comparison, the one-way ANOVA, Student’s t-test, and chi-squared tests were used. P < 0.05 was considered statistically significant.

## 3. Results

Homocysteine levels in both heterozygous and homozygous groups were higher than those of the control group (P < 0.05). RP was observed more frequently in patients with elevated homocysteine levels (P < 0.05, X2 = 14.51). There was no significant relationship in other parameters studied.

The demographic characteristics and studied parameters of patients are compared in Table 2.

**Table 2:** The demographic characteristics and laboratory findings of the control, heterozygous, and homozygous groups.

Characteristics	Control	Heterozygous	Homozygous	P value
Age (years)	36.04 ± 0.8	39.5 ± 12.5	44.1 ± 13.1	0.08
Homocysteine (mg/mL)	8.06 ± 2.4	9.16 ± 3.9	14.76 ± 3.0	0.00
Vitamin B12 (pg/mL)	399.25 ± 198.6	406.05 ± 171.11	340.18 ± 196.27	0.24
Folic acid (ng/mL)	11.98 ± 6.09	9.11 ± 4.72	9.27 ± 3.77	0.19
Platelet (103/uL)	256.93 ± 84.34	249.29 ± 76.02	253.46 ± 61.98	0.83
WBC* (103/mL)	7600 ± 2300	7787 ± 2270	7669 ± 2167	0.88
CRP**(mg/L)	8.4 ± 3.7	8.6 ± 3.6	7.6 ± 2.9	0.77

*White blood cell; **C reactive protein.

Homocysteine levels were higher than normal in 14 (33%) patients in the control group. In the MTHFR heterozygous group, 202 (73.5%) patients had high homocysteine levels; in the homozygous group, 39 (79.1%) patients. There was a significant difference between RP existence in MTHFR mutation groups (P < 0.05, X2 = 40.60). The relationships between homocysteine levels and MTHFR mutations are shown in Table 3.

**Table 3:** Homocysteine levels and MTHFR gene mutations of patients.

	Normal	High	Total	P and X2
Control	47 (77%)	14 (33%)	61 (100%)	P < 0.05X2 = 58.81
MTHFR heterozygous	73 (26.5%)	202 (73.5%)	275 (100%)	
MTHFR homozygous	13 (20.9%)	39 (79.1%)	52 (100%)	
Total	133 (34.3%)	255(65.7%)	388 (100%)	

The relationships between RP existence and MTHFR mutations are shown in Table 4. RP existence was more frequent in the MTHFR homozygous mutation group (P < 0.05).

**Table 4:** The existence of RP and MTHFR mutation.

	Positive	Negative	Total	P and X2
Control	9 (14.7%)	52 (85.3%)	61 (100%)	P < 0.05X2 = 15.97
MTHFR heterozygous	22 (8%)	253 (92%)	275 (100%)	
MTHFR homozygous	14 (26.9%)	38 (73.1%)	52 (100%)	
Total	45 (11.6%)	343 (88.4%)	388 (100%)	

When the relationships between homocysteine levels and RP existence are analyzed, 19 (6.9%) patients experienced RP although they had normal blood homocysteine levels. RP was not observed in 257 (93.1%) patients with normal blood homocysteine levels. In patients with high levels of homocysteine, 37 (33.0%) experienced RP, while 56 (67.0%) did not. Homocysteine levels and frequency of RP are shown in Table 5. The relationships between RP and homocysteine levels were analyzed using a chi-squared test. Similar to the results of the MTHFR mutation positive group, RP was observed more frequently in patients with elevated homocysteine levels (P < 0.05, X2 = 14.51).

**Table 5:** Homocysteine levels and frequency of RP.

Homocysteine levels	RP			P and X2	Positive	Negative	Total
	Normal	19 (6.9%)	257 (93.1%)		276 (100%)	P < 0.05X2 = 44.11
High	37 (33.0%)	75 (67.0%)	112 (100%)
Total	56 (14.4%)	332 (85.6%)	388 (100%)

## 4. Discussion

A significant relationship between plasma homocysteine levels and MTHFR gene mutations has been identified in this study. Furthermore, RP was more frequently observed in patients with hyperhomocysteinemia. Dietary folate intake and MTHFR gene polymorphisms are major contributing factors for plasma homocysteine levels [23].

The plasma level of homocysteine affects endothelial functions. Folic acid treatment may have beneficial effects on endothelial dysfunction by decreasing homocysteine plasma levels [24,25]. However, Pullin et al. demonstrated in their study that low-dose folate intake reduced homocysteine levels, but endothelial functions did not improve [26].

There are few studies associated with PRP and plasma homocysteine levels. Marasini et al. [22] conducted a study with 30 patients with systemic sclerosis, 12 patients with PRP, and 20 healthy patients as the control group. They inspected MTHFR mutations, homocysteine, von Willebrand factor (vWF), folate, and vitamin B12 levels. Plasma homocysteine levels were found to be higher in patients with secondary RP than patients with primary RP and healthy controls. Plasma folate and vitamin B12 levels were lower and vWF levels were higher in the SRP group compared to PRP or control groups. Levy et al. worked with patients who had primary RP and systemic sclerosis with RP; homocysteine levels were found significantly higher in patients with primary RP and systemic sclerosis patients rather than the control group, but there was no significant difference between primary and secondary groups with RP. Plasma folate levels of patients with primary RP were lower than in the secondary RP and control groups in the same study [27]. Similar results were obtained in the study by Al-Awami et al. [28]. Forty-two patients with connective tissue disorders such as SLE, mixed connective tissue disease, or systemic sclerosis and 26 patients with PRP were enrolled; homocysteine levels were analyzed in patients and compared with a control group of 45 healthy volunteers. No difference was found between homocysteine levels of patients with primary and secondary RP, but the average homocysteine levels in both groups were found to be higher than those of the control group. There were no differences in folate or vitamin B12 levels among the groups. Caramaschi et al. reported 60 patients with systemic sclerosis and secondary RP; the mean homocysteine levels were found to be significantly higher than in the control group; homocysteine levels were significantly positively correlated to vascular degeneration in the nail bed [29]. Czupryniak et al. reported 21 patients with end-stage renal disease (ESRD) who were on a hemodialysis program, 10 of whom had RP but 11 did not, and compared them to 9 healthy controls. Serum homocysteine levels were found to be significantly higher in patients with RP compared to patients without RP [30]. Cheng et al. reported a study with 34 patients having systemic lupus erythematosus and 20 healthy controls [31]. Eleven of the patients had RP. Homocysteine levels were found to be higher in SLE patients with RP than in control patients without RP and healthy controls. All these data are consistent with the results of this study.

Regardless of the existence of MTHFR mutation, frequency of RP was found to be 11.5% in the entire study population. This is a higher prevalence of RP compared to the general population. This may be caused by female patient dominance in the study, and possibly also because the majority of female patients had MTHFR mutations. Patients having homozygous MTHFR mutations have higher levels of homocysteine, and homocysteinemia in these patients more frequently causes endothelial dysfunction and eventually higher rates of RP occurrence.

In conclusion, RP was more frequently observed in groups with MTHFR mutations and hyperhomocysteinemia. Serum homocysteine levels in patients with RP may be helpful for diagnosis. Gene mutation screening will be useful in these patients. If mutations are found, these patients must be assessed for early treatment of hyperhomocysteinemia, and early intervention with antithrombotic or antiaggregants must be considered.

There were some limitations of the present study. There was female dominance in the study population, as some male patients who were eligible did not want to participate in the study. Most of the participants included in the study were female patients admitted to the gynecology and obstetrics clinic. Due to recurrent miscarriage, the numbers of patients evaluated for the MTHFR gene mutation in gynecology and obstetrics clinics are more frequent. Because of this, heterozygous and control groups have more females than male patients. However, when only the males are considered for MTHFR gene mutations, 17.16% were negative, 72.93% were heterozygous, and 9.9% were homozygous. These rates were 5.88%, 56.47%, and 37.64% respectively in the females.

## Acknowledgment

The authors of this study sincerely thank Dr Murat B KÜÇÜKAY for his support in article retrieval, revision, and translation of the document.

## References

[ref1] (2005). Pathogenesis of Raynaud’s phenomenon. Rheumatology.

[ref3] (2017). ESVM guidelines – the diagnosis and management of Raynaud’s phenomenon. Vasa.

[ref5] (2010). #8217;s phenomenon: A review. Autoimmunity Reviews.

[ref8] (1990). Serum endothelin-1 concentrations and cold provocation in primary Raynaud’s phenomenon. Lancet.

[ref10] (1999). Effect of nitric-oxide-generating system on microcirculatory blood flow in skin of patients with severe Raynaud’s syndrome: a randomised trial. Lancet.

[ref12] (1990). Deficiency of calcitonin gene-related peptide in Raynaud’s phenomenon. Lancet.

[ref14] (1982). The TH. The Journal of Rheumatology.

[ref16] (1985). Increased prostacyclin metabolites and decreased red cell deformability in patients with systemic sclerosis and Raynaud’s syndrome. Prostaglandins, Leukotrienes, and Medicine.

[ref18] (1976). Abnormal blood viscosity in Raynaud’s phenomenon. Lancet.

[ref20] (1997). Geographic variation in the prevalence of Raynaud’s phenomenon: a 5 region comparison. The Journal of Rheumatology.

[ref23] (2015). risk factors and associations of primary Raynaud’s phenomenon: systematic review and meta-analysis of observational studies. BMJ Open.

[ref25] (2005). #8217;s phenomenon in a healthy Turkish population. Clinical Rheumatology.

[ref27] (2008). Prevalence of Raynaud’s phenomenon in healthy Turkish medical students and hospital personnel. Rheumatology International.

[ref29] (2001). Polymorphisms in the methylentetrahydrofolate reductase gene: clinical consequences. American Journal of Pharmacogenomics.

[ref31] (2019). Folic acid and vitamin B12 administration in CKD, why not. Nutrients.

[ref34] (2001). MTHFR gene polymorphism, homocysteine and cardiovascular disease. Public Health Nutrition.

[ref36] (2002). MTHFR 677C-T polymorphism and risk of coronary heart disease. JAMA.

[ref38] (1995). -methylenetetrahydrofolate reductase as a cause of mild hyperhomocysteinemia. Thermolabile.

[ref40] (1996). Relation between folate status, a common mutation in methylenetetrahydrofolate reductase, and plasma homocysteine concentrations. Circulation.

[ref42] (1991). Hyperhomocysteinemia: an independent risk factor for vascular disease. The New England Journal of Medicine.

[ref44] (1999). Endothelial dysfunction by acute hyperhomocyst(e)inaemia: restoration by folic acid. Clinical Science.

[ref46] (2000). Homocysteine concentration in primary and systemic sclerosis associated Raynaud’s phenomenon. The Journal of Rheumatology.

[ref49] (1999). Oral folate enhances endothelial function in hyperhomocysteinaemic subjects. European Journal of Clinical Investigation.

[ref51] (2017). The importance of folate, vitamins B6 and B12 for the lowering of homocysteine concentrations for patients with recurrent pregnancy loss and MTHFR mutations. Reproductive Toxicology.

[ref54] (2004). homocysteine metabolism and CVD. Proceedings of the Nutrition Society.

[ref56] (2001). Optimization of dietary folate or low-dose folic acid supplements lower homocysteine but do not enhance endothelial function in healthy adults, irrespective of the methylenetetrahydrofolate reductase (C677T) genotype. Journal of American College of Cardiology.

[ref58] (1999). Elevated homocysteine levels in patients with Raynaud’s syndrome. The Journal of Rheumatology.

[ref60] (2002). Homocysteine levels in patients with Raynaud’s phenomenon]. Homozysteinspiegel von Patienten mit Raynaud-Phänomen [.

[ref64] (2007). Correlation between homocysteine plasma levels and nailfold videocapillaroscopic patterns in systemic sclerosis. Clinical Rheumatology.

[ref67] (2005). #8217;s phenomenon and endothelial dysfunction in end-stage renal disease patients treated with hemodialysis. Kidney &amp; Blood Pressure Research.

[ref69] (2002). Elevated homocysteine levels in patients with Raynaud’s phenomenon secondary to systemic lupus erythematosus. Clinical Rheumatology.

